# Development of a Quantitative Bead Capture Assay for Soluble IL-7 Receptor Alpha in Human Plasma

**DOI:** 10.1371/journal.pone.0006690

**Published:** 2009-08-19

**Authors:** Sylvie Faucher, Angela M. Crawley, Wendy Decker, Alice Sherring, Dragica Bogdanovic, Tao Ding, Michele Bergeron, Jonathan B. Angel, Paul Sandstrom

**Affiliations:** 1 National HIV and Retrovirology Laboratories, Public Health Agency of Canada, Ottawa, Canada; 2 Ottawa Hospital Research Institute, Ottawa, Canada; 3 Department of Biochemistry, Microbiology and Immunology, University of Ottawa, Ottawa, Canada; 4 Division of Infectious Diseases, Ottawa Hospital-General Campus, Ottawa, Canada; New York University School of Medicine, United States of America

## Abstract

**Background:**

IL-7 is an essential cytokine in T-cell development and homeostasis. It binds to the IL-7R receptor, a complex of the IL-7Rα (CD127) and common γ (CD132) chains. There is significant interest in evaluating the expression of CD127 on human T-cells as it often decreased in medical conditions leading to lymphopenia. Previous reports showed the usefulness of CD127 as a prognostic marker in viral infections such as HIV, CMV, EBV and HCV. A soluble CD127 (sCD127) is released in plasma and may contribute to disease pathogenesis through its control on IL-7 activities. Measuring sCD127 is important to define its role and may complement existing markers used in lymphopenic disease management. We describe a new quantitative assay for the measurement of sCD127 in plasma and report sCD127 concentrations in healthy adults.

**Methodology/Principal Findings:**

We developed a quantitative bead-based sCD127 capture assay. Polyclonal CD127-specific antibodies were chosen for capture and a biotinylated monoclonal anti-CD127 antibody was selected for detection. The assay can detect native sCD127 and recombinant sCD127 which served as the calibrator. The analytical performance of the assay was characterized and the concentration and stability of plasma sCD127 in healthy adults was determined. The assay's range was 3.2–1000 ng/mL. The concentration of plasma sCD127 was 164±104 ng/mL with over a log variation between subjects. Individual sCD127 concentrations remained stable when measured serially during a period of up to one year.

**Conclusions/Significance:**

This is the first report on the quantification of plasma sCD127 in a population of healthy adults. Soluble CD127 plasma concentrations remained stable over time in a given individual and sCD127 immunoreactivity was resistant to repeated freeze-thaw cycles. This quantitative sCD127 assay is a valuable tool for defining the potential role of sCD127 in lymphopenic diseases.

## Introduction

Interleukin-7 (IL-7) is essential for the development and survival of human T cells [1]. The IL-7R is a heterodimeric receptor complex composed of the common cytokine receptor γ_c_ chain (CD132) found in several other cytokine receptors (IL-2R, -4R, -9R, -15R, and -21R) and the IL-7Rα chain (CD127), also a component of the Thymic Stromal Lymphopoietin (TSLP) receptor complex [2]–[5]. CD127 deficiency due to gene mutations in the CD127 gene results in severe combined immunodeficiency (SCID) in both mice and humans [6], [7]. Modulation of CD127 expression has been observed in a number of diseases [8]–[10]. We and others have demonstrated that significantly fewer CD8^+^ T cells express CD127 in HIV-infected individuals and this correlates with increased plasma viremia and prognostic markers such as CD4 depletion and markers of immune activation [11]–[17] The mechanism(s) for the loss of membrane-associated CD127 is an active area of investigation. We and others have also shown that IL-7 downregulates CD127 expression on CD8^+^ T-cells and CD4^+^ T-cells [16], [18], [19]. In addition to the membrane bound receptor, a soluble form of the CD127 (sCD127) can be generated by alternative splicing of mRNA transcripts encoding CD127. This results in a truncated polypeptide composed of the extracellular domain and a short 27 amino acid C-terminus encoded by the altered reading frame. [20], [21].

The expression of the alternatively spliced CD127 transcript was reported in healthy individuals [20] and increased expression has been described in acute lymphoblastic leukaemia (ALL) [22]. A mutation in the transmembrane domain of CD127 has been associated with the production of mRNA transcripts encoding sCD127 in multiple sclerosis patients [23], [24]. Soluble CD127 was initially detected in the supernatant of WI-26VA4 cells, a SV-40 transformed human lung epithelial cell line shown to release sCD127 using an IL-7 binding assay [25]. Carini et al. described an assay used to detect sCD127 in the culture supernatants of human CD8^+^ T-cells, however this involved the labour-intensive purification of sCD127 using an IL-7-conjugated affinity chromatography column followed by a CD127-specific ELISA [25]. As IL-7 and surface CD127 are important prognostic indicators in HIV infection, sCD127 may play a role in the pathogenesis of HIV and other diseases as well, as is the case with other soluble cytokine receptors. We report herein the development of a quantitative capture immunoassay for the measurement of the sCD127 chain and assess its concentration and stability in the plasma of healthy individuals.

## Results

### Assay characteristics

**Figure 1:**
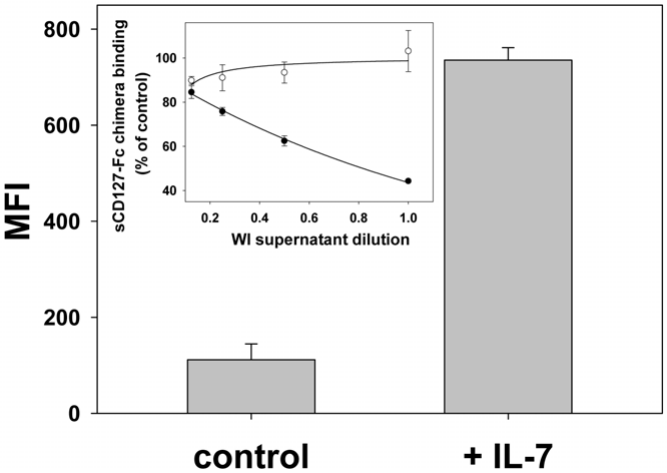
Detection of native sCD127 using the sCD127 capture bead assay. Detection of native sCD127 in the culture supernatant of unstimulated (Ctrl) and IL-7-stimulated WI-26VA4 cells using the sCD127 capture bead assay. Insert: Specificity of the sCD127 capture bead assay for native sCD127. Inhibition of recombinant sCD127-Fc chimera binding to sCD127 assay's capture antibody by native sCD127. In these experiments, recombinant sCD127-Fc chimera binding to sCD127 capture antibody conjugated to bead was detected using a Fc-specific detection antibody. The residual binding is expressed as the percent of the fluorescence signal (MFI) in presence over the signal in absence of native sCD127 from unstimulated (empty circles) or IL-7-stimulated (filled circles) WI-26VA4 cell culture supernatants.

Since this assay was based on capture antibodies that were developed to be specific for the extracellular domain of a recombinant form of CD127, the assay reactivity toward the native form of sCD127 was first established. The human WI cell line is well characterized for the shedding of the soluble form of CD127 and was used as a source of native sCD127. Soluble CD127 released by WI cells after a 24 hour stimulation with IL-7 was detected by the assay anti-CD127 capture antibody (Fig. 1). The assay specificity was then assessed using WI shed sCD127 as a competing ligand to anti-CD127 capture antibody. In this experiment, anti-CD127 antibody coated beads were incubated with recombinant sCD127-Fc chimera and an excess of native sCD127 from WI supernatant. The residual binding of the recombinant sCD127-Fc chimera was quantified using an Fc-specific biotinylated antibody. The native sCD127 was able to inhibit the binding of the recombinant sCD127-Fc chimera in a dose dependent manner and competed out 60% of the recombinant receptor when undiluted WI cell culture supernatant (containing 309 ng/mL of native sCD127) was used (Fig. 1 insert).

**Figure 2:**
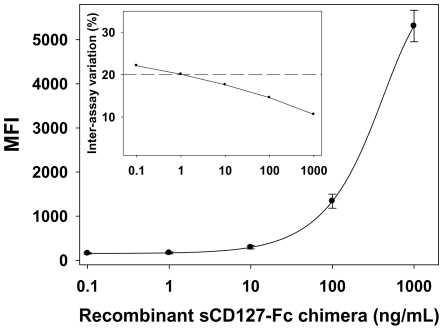
Calibration curve of the sCD127 capture bead assay. Recombinant sCD127-Fc chimera was serially diluted from 0.1 to 1000 ng/mL in assay diluent (Materials and Methods) and used as the assay's calibrator. The recombinant sCD127 was captured on beads conjugated with sCD127 polyconal antibodies. Bound sCD127 was detected with a biotinylated monoclonal anti-CD127 and streptavidin-PE. The curve was resolved using a 5-parameter logistic equation (MasterPlex QT software). Insert: Standard curve inter-assay variation from 12 assays done in duplicates. The curve's range was 3.2–1000 ng/mL.

The sCD127 capture assay was calibrated with purified recombinant CD127-Fc chimera from 0.1 to 1000 ng/mL (Fig. 2). Curves generated from 12 separate assays from 2 different calibrator's batches and spread over a 9 month-period showed a mean variation in fluorescence signal of 17% (range 10.6–22.1%) over the entire calibrator curve (Fig. 2, insert). Inter-assay precision was established by replicated measures of two samples of known concentration in 7 assays and showed a mean of 322.1±36.5 ng/mL (CV of 11.3%, n = 14) and 1006.9±162.8 ng/mL (CV of 16.2%, n = 13). One sample was tested in replicates of 14 in 3 assays to assess intra-assay precision and showed a mean concentration of 256±16.4 ng/mL with a variation of 6.3% (range CV 5.2–8.1%). The minimum detectable dose, determined as the mean of 30 replicates of the zero calibrator plus 2 SD was 2.6 ng/mL. The lowest measurable concentration of sCD127 was 3.2 ng/mL (CV = 18%).

Assay linearity was assessed using a two-fold serial dilution (1/10 to 1/40) of 3 plasma samples with assay diluent. The mean recovery was 109.5%±19.2% (sCD127 323 ng/mL), 109.5%±20.2% (sCD127 140 ng/mL) and 100.9%±6.8% (sCD127 270 ng/mL).

The potential interference of IL7 with the measurement of sCD127 was assessed in a series of samples containing a fixed concentration of sCD127 and increasing amount of recombinant IL-7. The presence of IL7 did not interfere with the detection of sCD127 even when IL-7 was present in excess at a molar ratio of 10 to 1 (data not shown).

### sCD127 in healthy adults

**Figure 3:**
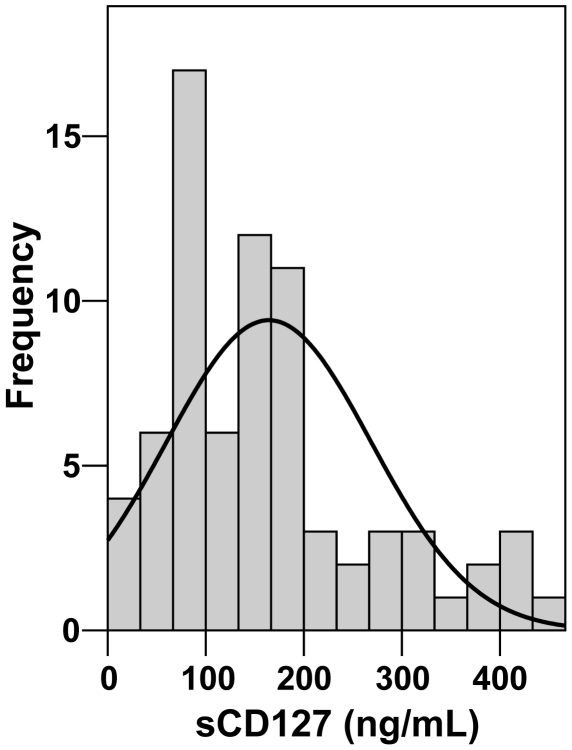
Soluble CD127 concentration distribution in healthy adults and normal Gaussian curve. Plasma concentrations of sCD127 (n = 74) were determined using a sCD127 capture bead assay. Each bar represents the number of cases for each interval of sCD127 values and the curve represents the predicted normal distribution. The mean sCD127 was 164.3±104.5 ng/mL.

Volunteers (n = 74) from our facility were recruited for the determination of sCD127 concentration in healthy adults. Soluble CD127 concentrations ranged from <32 ng/mL to 462.6 ng/mL (mean 164.3±104.51 ng/mL) (Fig. 3). Four samples among the 74 tested (5.4%) had concentrations of sCD127 below the detection limit of the assay. The data range observed from this group of subjects did not obey a predicted Gaussian distribution (Shapiro-Wilk test, W = 0.912, P<0.001). The concentrations of sCD127 were analysed for correlation with gender (38% male, 62% female) and age (male age 42±10; female age 45±15; range 21–65 years) from 29 individuals for which data were available and showed no statistically significant correlation with either parameter.

### Individual plasma sCD127 variation over time

**Figure 4:**
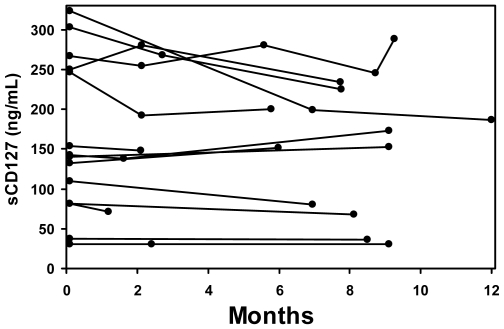
Serial measurements of sCD127 concentrations in plasma of healthy individuals. The concentration of sCD127 was measured in the plasma of repeat donors (n = 15) tested 2–5 times over a one-year period. Individual concentrations of sCD127 remained within 14.1±11.3% of their original values (range 0–42%).

The concentrations of sCD127 were measured in repeat donors (n = 15) tested 2–5 times during a 12-month period (total samples = 39). The sCD127 concentrations remained stable over time with a mean variation of 30.6±35.5 ng/mL (range 0–136.8) (Fig. 4). This variation represented an average of 14.1±11.3% (range 0–42.4%) from the initial sCD127 measurement.

### Effect of freeze-thawing on sCD127 detection

A stability study was undertaken to monitor sCD127 immunoreactivity robustness to repeat freezing process. Soluble CD127 concentrations were assessed in plasma samples from 5 individuals (sCD127 concentrations range 100 to 270 ng/mL). The sCD127 concentrations were stable in all samples for up to 4 cycles of freeze-thaw with a mean variation of 4.6% (range 0.5–13%) (Table 1).

**Table 1:** Effect of freeze-thawing on sCD127 immunoreactivity.

Samples	Freeze-thaw cycles		CV^b^
	1	4	%
1	270.0±6.4^a^	257.3±19.7	3.4
2	99.8±1.9	100.5±6.1	0.5
3	185.3±1.3	154.2±7.2	13.0
4	269.2±13.7	265.4±3.0	1.0
5	246.3±11.2	228.9±3.0	5.2

a sCD127 concentration in ng/mL (mean±SD).

b CV calculation: SD/mean ×100.

## Discussion

There is increasing interest in the quantification of sCD127 in human plasma mainly motivated by the pivotal role of the IL-7/IL-7R pathway in an increasing number of diseases. We and others have measured sCD127, in vitro and in plasma [19], [25]. Carini et al. developed an immunosorbent assay combining CD127 capture and IL-7 detection to quantify sCD127 in culture supernatants. This assay had limited sensitivity and required a substantial amount of sample processing. We have previously reported semi-quantitative evaluation of sCD127 in human plasma by Western blot, however this does not constitute a practical approach for large scale studies. We have described here the development of a sensitive and reproducible bead-based assay for the quantitative measurement of human sCD127 in plasma and report the sCD127 concentrations in healthy individuals.

The sCD127 assay detection range was 3.2–1000 ng/mL and showed sufficient sensitivity to measure sCD127 in 95% of individuals tested. Matrix effects were avoided by a 10-fold dilution of plasma samples and the reconstitution of the calibrator in a negative human plasma control. Since the present assay included only samples from healthy individuals, potential interference in pathological plasma samples cannot be excluded. The sCD127 appears to be a robust protein marker as repeated sample freeze-thaw cycles and prolonged plasma storage (data not shown) did not affect detection. This suggests that the sCD127 would be a valuable marker for retrospective studies using archived samples.

The mean concentration of sCD127 in healthy adults was 164.3 ng/mL and appears to remain stable over time in any given individual however a wide range of concentrations was observed between individuals (below 32 ng/mL to over 400 ng/mL). This variation represents more than a log difference in the population of healthy individuals studied here. A very recent report by Blom-Potar et al. [26] investigated the levels of sCD127 in human plasma using an in-house ELISA. Although not representative of a population, the mean concentration of sCD127 determined from the 6 healthy subjects in this study's control group (23.6 ng/mL) was much lower than the mean sCD127 concentration reported here. This difference is likely explained by the different combination of sCD127 clones used in the ELISA as well as to the very small number of individuals tested.

It is interesting to note that sCD127 concentrations did not correlate with percentages of CD4+CD127+ or CD8+CD127+ T-cells in peripheral blood (data not shown). Whether or not the concentration of sCD127 varies during episodes of common innocuous infections or in disease states has not been explored here and remains to be determined.

Despite previous reports of detecting sCD127 in the culture supernatants of CD8+ T-cells by Western blot [19], this assay has failed to detect sCD127 in cultures of human CD8^+^ T cells. However, the assay can detect sCD127 in WI-26VA4 cell culture supernatants. To date, there has only been one report of a semi-quantitative method for detecting sCD127 in CD8+ T-cell culture supernatants, in which sCD127 is first purified using an IL-7-conjugated affinity chromatography column [25]. This is an ongoing area of investigation as the detection of sCD127 *in vitro* would benefit greatly from a quantitative sCD127 assay.

As reported previously, a limited amount of IL-7 is found in plasma of healthy individuals [17]. In the present study, the mean plasma concentration of IL-7 in 29 individuals was low (2.2 pg/mL, range 0.2–10.5 pg/mL) and no correlation was observed with circulating levels of sCD127 (data not shown). Attempts were made to detect sCD127-IL-7 complexes in plasma and WI-26VA4 culture supernatant using the present assay sCD127 capture beads and a non-neutralizing anti-IL-7 detection monoclonal antibody. Both plasma and WI supernatant tested contained sCD127 and IL-7 that could be measured using sCD127 and IL-7 specific assays. However, no sCD127-IL-7 complexes were detected in the plasma or in WI cell culture supernatants using the sCD127 capture beads and IL-7 detection antibody (data not shown). Despite a previous report demonstrating the detection of sCD127-IL-7 complexes using an ELISA [25], our approach failed to detect such complexes. Capture of the complexes via polyclonal antibodies to IL-7 or non-neutralizing IL-7 monoclonal antibody is presently being investigated. Attempt to measure IL-7 interference in the detection of sCD127 was done by the addition of exogenous recombinant human IL-7 to a constant concentration of recombinant sCD127. Our assays demonstrated that the presence of IL-7 did not interfere with the resulting sCD127 concentrations. If proven to be the case for plasma sCD127 and IL-7, this assay would provide a useful approach for total sCD127 measurements.

These data support the use of this assay to reliably and accurately quantify human sCD127 in plasma. In addition, the mechanism by which the receptor is secreted is an active area of interest. Potential roles for the sCD127 included the modulation of circulating IL-7, thereby either limiting IL-7-related activities or acting as a chaperone and extending the cytokine's half-life. IL-7 is critical for T-cell development and is an important factor for T-cell homeostasis and memory development. The presence of increased concentrations of sCD127 in the periphery could pose a threat for the delivery of an adequate amount of IL-7 signals to cells known to express CD127 such as naïve and memory CD4^+^ and CD8^+^ T-cells. The resulting consequences would likely be reduced IL-7 signaling and function such as decreased expression of anti-apoptotic molecules, less proliferation, halted cell differentiation and reduced anti-viral/tumour functions. In addition, peripheral regulatory T-cells (Tregs), which typically do not express, or express low levels of CD127 [27]–[29] might be spared from IL-7 deprivation [30]. This could impact on the ratio of effector T-cells and Tregs and alter the fine balance governing the immune response [31]. Studies of the levels of circulating sCD127 in autoimmune disorders have yet to be done to establish the potential relation of sCD127 to other markers of autoimmunity.

Several soluble cytokine receptors have been shown to be of prognostic value in a number of diseases and have also been developed as therapeutic agents in the treatment of a number of inflammatory diseases [32]. For example, soluble IL-2Rα (sIL-2Rα), a marker of immune activation in diseases including reumatoid arthritis, and various cancers, has proved useful in monitoring disease progression and early detection of recurrent disease [33]. Serum sIL-2Rα concentrations in patients with leukemia and autoimmune diseases can reach concentrations up to 100 fold those found in normal individuals [34]. Soluble IL-2Rα has been associated with leukemia disease progression and poor prognosis in children with Hodgkin's disease [35], [36]. Alleles of the IL-2Rα gene region have been linked to susceptibility and risk to multiple sclerosis and Type 1 diabetes and these allelic variants independently correlate with plasma sIL-2Rα concentrations [37]. Increase in soluble TNF receptors can inhibit TNF signaling and has been shown to prevent TNF-mediated tumour lysis [38]. In its soluble form, the IL-6 receptor subunit gp130 inhibits the pro-inflammatory activity of IL-6 [39]. Pre-complexed IL-15-sIL-15Rα increases NK and CD8^+^ T-cell proliferation and anti-tumour activity up to 50 times compared to IL-15 alone [40]–[42].

A characterization of sCD127 function and relevance in HIV infection is forthcoming. The expression of CD127 is decreased in infections with latent viruses such as EBV and CMV as well as in chronic viral infections such as HIV and hepatitis-C virus (HCV) [16], [43]–[45]. It has been shown that CD127 expression is decreased in response to IL-7 *in vitro* while the release of sCD127 increases [19]. The relevance of sCD127 in HIV infection is of particular interest, as plasma concentrations of IL-7 are increased in infected individuals [46].

We specifically developed a bead-based assay as this format allows for multiplex analysis. Future work involving the addition of other cytokines and/or soluble cytokine receptors would enhance the coverage of other pathways and hence, would offer a more systemic approach to the study of the cytokine network and better disease prognosis.

The development of a quantitative assay specific to human sCD127 will be a valuable tool in determining whether sCD127 concentrations are altered in diseases in which the expression of CD127 is modulated and may assist in investigations of receptor function. Given that IL-7 is being studied in HIV and cancer treatments [47], [48], measuring sCD127 may be important when assessing the outcomes of these therapies.

## Materials and Methods

### Blood collection

All research conducted using blood from human subjects was approved by the Ottawa Hospital Research Ethics Board on the basis of written informed consent. A total of 101 plasma samples were obtained from 74 in-house healthy donors. Whole blood was collected in K_2_EDTA-containing Vacutainer Tubes (BD Biosciences) and processed within 2 hours from collection time. Repeat donors (n = 15) were bled 2–5 times over a period of one year.

### Cell cultures

The WI-26VA4 human fibroblast cell line (WI, ATCC) was cultured in RPMI culture medium supplemented with 10% FCS and penicillin/streptomycin until approximately 80% confluent. Then the cells were further incubated for 24 or 48 hrs with or without recombinant human IL-7 (BD Biosciences) at 37°C and 5% CO_2_. Following the centrifugation of cell pellets, culture supernatants were collected and stored at −80°C.

### Soluble CD127 capture assay

The sCD127 bead-based capture assay was developed after testing capture and detection antibodies from several commercial sources (BD Biosciences, Beckman Coulter, eBioSciences, BioLegend, R&D Systems, Sigma-Aldrich). Antibodies were selected for their ability to capture both the native form of human sCD127 and a recombinant sCD127-Fc chimera (R&D Systems) used as the assay's calibrator. Goat anti-human CD127 polyclonal antibodies (R&D Systems) were covalently coupled to carboxylated-modified fluorescent beads (Luminex, TX) using carbodiimide and N-hydroxysuccinimide as described before [49]. The antibody-coupled beads were stable for at least one year when kept in the dark at 4°C. The lyophilized human sCD127-Fc chimera was reconstituted in phosphate buffer saline (PBS, Canadian Life Technologies Inc., Canada) containing 0.1% BSA (Sigma-Aldrich) and kept frozen at −80°C. The calibrator was diluted in assay diluent consisting of PBS containing 0.05% Tween-20 (Sigma-Aldrich), 1% BSA, 0.05% NaN_3_ (Sigma-Aldrich), and 0.9 mg/mL EDTA (Sigma-Aldrich). The calibrator curve was prepared in assay diluent containing a 10-fold dilution of a human plasma (negative control) selected for its undetectable levels of sCD127. Fifty µL of 10 fold diluted plasma samples in assay diluent were incubated with the anti-CD127-coated beads (3000/well) for 2 hrs at room temperature. After 3 washes with PBS containing 0.05% Tween and 0.9 mg/mL EDTA, the plate was incubated with a biotinylated mouse anti-human CD127 antibody (BD Biosciences) diluted in the assay diluent for 1 hr at room temperature. The plate was washed as before and then incubated with streptavidin-PE (Molecular Probes) for 10 minutes at room temperature. After 3 washes the samples were analysed on a Luminex-100 System using the median fluorescence intensity (MFI) of 100 events as the read-out. The bead analysis was done using the MiraiBio software (MasterPlex CT, version 1) and the standard curves were generated using a 5-parameter logistic curve fitting equation (MasterPlex QT software, version 4, MiraiBio). The assay background was 162.4±14.9 MFI (CV 9.2%, n = 30) and the limit of detection was set at 192.2 MFI (bkg+2 SD).

### Competitive binding to anti-CD127 conjugated beads

The culture supernatant of IL-7-stimulated WI cells is known to contain sCD127 [20] and was used as a source of native sCD127 in these experiments. Two fold dilution of WI cell culture supernatant (undiluted, 1/2, 1/4, 1/8) from unstimulated and IL-7-stimulated cultures were incubated with a fixed concentration of recombinant sCD127-Fc chimera (0.5 ng/mL) and the anti-sCD127 conjugated beads for 2 hours. Residual recombinant sCD127-Fc chimera binding to the beads was measured using a biotinylated anti-human Fc-specific monoclonal antibody (Jackson ImmunoResarch Laboratories) and was detected with streptavidin-PE. The residual binding was expressed as (MFI in presence of competitor – background)/(MFI in absence of competitor – background) ×100.

### IL-7 interference assay

Soluble CD127 was determined in samples containing a fixed concentration of recombinant sCD127 (5 ng/mL) in presence of increasing amount of recombinant IL-7 (BD Biosciences) (range 0.003–15 000 pg/mL; 0–10 IL-7/sCD127 molar ratio). The sCD127 was measured as described above.

### sCD127 freeze-thaw stability

Plasma samples were subjected to 4 cycles of freeze-thaw under conditions that would mimic handling conditions of plasma used in repetitive testing. Plasma aliquots of 100 μL were frozen at −80°C, thawed and remained at room temperature for 1 hour. The cycle was repeated 4 times and the concentration sCD127 was measured.

### Statistical analysis

Values are expressed as mean±SD. Correlations were determined using the Pearson correlation coefficient test and group comparisons were analysed with the Student's *t*-test using the Sigma-Plot software program (version 11, Systat software). Distribution and normality analysis were generated using the Shapiro-Wilk test (SPSS software package, version 12 for Windows).
